# Targeted Screening for Chronic Q Fever, the Netherlands

**DOI:** 10.3201/eid2807.212273

**Published:** 2022-07

**Authors:** Daphne F.M. Reukers, Pieter T. de Boer, Alfons O. Loohuis, Peter C. Wever, Chantal P. Bleeker-Rovers, Arianne B. van Gageldonk-Lafeber, Wim van der Hoek, Aura Timen

**Affiliations:** National Institute for Public Health and the Environment, Bilthoven, the Netherlands (D.F.M. Reukers, P.T. de Boer, A.B. van Gageldonk-Lafeber, W. van der Hoek, A. Timen);; Q-Support Foundation, ’s-Hertogenbosch, the Netherlands (A.O. Loohuis);; Radboud University Medical Center, Nijmegen, the Netherlands (A.O. Loohuis, C.P. Bleeker-Rovers);; Jeroen Bosch Hospital, ’s-Hertogenbosch (P.C. Wever);; Vrije Universiteit, Amsterdam (A. Timen)

**Keywords:** Q fever, targeted screening, chronic Q fever, Coxiella burnetii, bacteria, screening program, aneurysm, vascular disease, heart valve disease, immunocompromised patient, general practitioner, cost effectiveness, the Netherlands

## Abstract

Early detection of and treatment for chronic Q fever might prevent potentially life-threatening complications. We performed a chronic Q fever screening program in general practitioner practices in the Netherlands 10 years after a large Q fever outbreak. Thirteen general practitioner practices located in outbreak areas selected 3,419 patients who had specific underlying medical conditions, of whom 1,642 (48%) participated. Immunofluorescence assay of serum showed that 289 (18%) of 1,642 participants had a previous *Coxiella burnetii* infection (IgG II titer >1:64), and 9 patients were suspected of having chronic Q fever (IgG I y titer >1:512). After medical evaluation, 4 of those patients received a chronic Q fever diagnosis. The cost of screening was higher than estimated earlier, but the program was still cost-effective in certain high risk groups. Years after a large Q fever outbreak, targeted screening still detected patients with chronic Q fever and is estimated to be cost-effective.

Approximately 2% of patients with symptomatic or asymptomatic *Coxiella burnetii* infections show development of chronic Q fever (*1*). Chronic Q fever can manifest itself even years after the initial infection, mainly as endocarditis or vascular infection. The main risk factors for development of chronic Q fever are heart valve disorders, aortic aneurysms, vascular prosthesis, or an immunocompromised state. Chronic Q fever can cause potentially life-threatening complications and has a high mortality rate (*2*). Therefore, timely detection and treatment for patients who have chronic infections are essential.

A large Q fever outbreak that occurred in the Netherlands during 2007–2010 had >4,000 reported acute Q fever cases in humans and an estimated total number of 50,000 *C. burnetii* infections, mostly originating from dairy goat farms that experienced Q fever‒induced abortion waves (*3*). In the years after the outbreak, several hospitals in the most affected regions undertook small screening studies in specific risk groups for early detection of chronic Q fever (*4*–*7*). These studies showed that there were still undiagnosed chronic Q fever patients in these risk groups. Identifying these undiagnosed patients can provide major health benefits by reducing complications and deaths. A recent model-based study from the Netherlands estimated that targeted screening of patients who had risk factors in regions that had previous outbreaks, was cost-effective (*8*). After a strong appeal from Q fever patients and the involved physicians, a screening program was launched in the Netherlands 10 years after the Q fever outbreak.

This one-time targeted chronic Q fever screening program was implemented in general practitioner practices because of 3 factors. First, because these practices have smaller catchment areas, regions with previous outbreaks can be demarcated in more detail. Second, because these practices in the Netherlands have complete and up-to-date electronic files of their patients, all target groups can directly be selected without the need for patient files from different hospitals and medical specialists. Third, patients in the Netherlands have their regular check-ups often with their general practitioner after receiving specialist care. We report results from the early phase of this screening program and provide an update of the previously conducted cost-effectiveness analysis.

## Methods

### Patient Selection

A chronic Q fever screening program began during 2019 in general practitioner practices located in various high-risk areas (incidence >50 acute Q fever cases/100,000 persons or near a farm that had Q fever‒induced abortion waves during the outbreak of 2007–2010) across the Netherlands. Medical risk factors for chronic Q fever after acute infection are well described (*9*), and a list of International Classification of Primary Care (ICPC) codes was compiled with consensus from different experts (Table 1). Furthermore, although studies have shown that male patients have a higher risk for *C. burnetii* cardiovascular infection, we undertook the screening in both male and female patients because of ethics considerations (*10*,*11*).

**Table 1:** ICPC codes used for the selection of patients to be invited for participation in the chronic Q fever screening program, the Netherlands*

ICPC code	Description
K99.01	Aortic aneurysm
K83	Nonrheumatic valve disease
K73	Congenital malformation(s) of the cardiovascular system
K71	Acute rheumatism/rheumatic heart disease
B73	Leukemia
B74	Other malignancy of the blood/lymphatic system
B90	HIV infection
D94	Ulcerative colitis/chronic enteritis (regionalis)
L04A	Use immunosuppressants (excluding corticosteroids) in the past 12 months

*ICPC, International Classification of Primary Care.

A standard general practice that had 1 general practitioner in the Netherlands had on average 2,095 patients, and it was estimated that applying the selection list of ICPC codes would yield ≈80 high-risk patients/standard practice. Participating general practitioner practices (1 practice might have several general practitioners) selected patients eligible for screening with the selected ICPC codes (Table 1). These patients received information about chronic Q fever screening and were invited to visit a nearby center to provide a blood serum sample, either at the general practitioner practice or at a local blood drawing location in the residential area. A trusted third party was hired to support the general practitioners with sending out the invitations.

### Diagnostics

All samples were tested by laboratory technicians at Jeroen Bosch Hospital (‘s Hertogenbosch, the Netherlands), who used an immunofluorescence assay (IFA) to detect antibodies against *C. burnetii*. An IgG II titer >1:64 was considered evidence of a past *C. burnetii* infection, and an IgG I titer >1:512 was considered a positive screening test result for suspected chronic Q fever. We sent serologic results to the general practitioner, who would refer patients to a clinical center that had expertise in chronic Q fever in case of a positive screening test result. In these centers, a definite diagnosis was based on follow-up testing, medical examination, and radiologic imaging findings (transthoracic echocardiography or a positron emission tomography scan) according to the Netherlands consensus guidelines on chronic Q fever diagnostics (*9*). This guideline classifies the probability of having chronic Q fever as proven, probable, and possible. One of the criteria in these guidelines is an IFA titer >1:1,024 for IgG against *C. burnetii* phase I.

### Statistical and Cost-Effectiveness Analysis

We compiled patient characteristics and antibody test results with descriptive statistics. We calculated seroprevalence per general practitioner, as well as the number and percentage of suspected chronic Q fever patients per general practitioner. Previously, a health-economic decision model was developed to estimate the cost-effectiveness of the screening program (*8*). Results collected in our study were used to update the cost-effectiveness analysis. This use concerned the seroprevalence rate (i.e., prevalence of past *C. burnetii* infections), the proportion of seropositive patients receiving a definite diagnosis of chronic Q fever by risk group (cardiovascular or immune-related), and the screening costs per patient. The previous cost-effectiveness analysis envisaged a hospital-based screening program implemented in routine care by using a 2-step testing scheme, first with the ELISA, followed by the IFA test if the test result was positive. The rationale behind analysis was that the ELISA is an automated test available in any hospital, but the IFA is only conducted in a few specialized hospital laboratories. However, as mentioned, the actual screening program was implemented in general practitioner practices, and patients were directly tested by using the IFA because this test is considered to be the reference test, given its higher sensitivity.

## Results

### Participants

**Figure 1:**
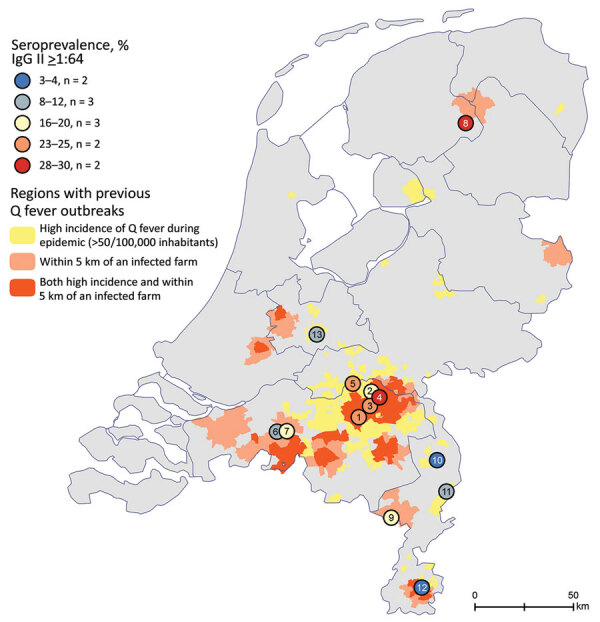
Locations of participating general practices (numbers in circles) in the Netherlands and seroprevalence rates for chronic Q fever measured in study of targeted screening program to detect chronic Q fever. Colors indicate areas with high incidence of acute Q fever patients or areas near an infected farm that had abortion waves during the outbreak of 2007–2010.

A total of 13 general practitioner practices located in regions that had a high incidence of Q fever during the outbreak or a location near an infected goat farm participated in the study (Figure 1) during May 2019‒December 2020. All general practitioner practices except 1 were located in the southern part of the Netherlands, where most Q fever cases were reported during the outbreak. The general practitioners invited 3,419 eligible patients, of whom 1,642 (48%) participated (Table 2). The largest practice invited 477 eligible patients, and the smallest practice invited 108 patients.

**Table 2:** Results of a targeted screening program to detect chronic Q fever, the Netherlands*

GP practice†	Province	No. eligible patients‡	Study participants, no. (%)	Seroprevalence (IgG II titer >1:64), no. (%)	No. suspected of having chronic Q fever (IgG I titer >1:512)
1	NB	358	216 (60)	51 (24)	0
2	NB	250	108 (43)	18 (17)	0
3	NB	477	255 (53)	58 (23)	2
4	NB	267	160 (60)	48 (30)	2
5	NB	144	84 (58)	21 (25)	1
6	NB	381	183 (48)	22 (12)	1
7	NB	108	58 (54)	9 (16)	0
8	FR	124	40 (32)	11 (28)	0
9	LI	308	143 (46)	28 (20)	2
10	LI	376	147 (46)	4 (3)	0
11	LI	239	110 (46)	9 (8)	0
12	LI	134	53 (40)	2 (4)	1
13	UT	253	85 (34)	8 (9)	0
Total	NA	3,419	1,642 (48)	289 (18)	9

*GP, general practitioner; FR, Friesland; LI, Limburg; NA, not applicable; NB, North-Brabant; UT, Utrecht. †Corresponding to numbers in Figure 1. **‡Eligible patients are patients who had increased risk for development of chronic Q fever after infection with *Coxiella burnetii* (see ****Table 1**** for specified inclusion criteria).**

### Seroprevalence

The average seroprevalence of IgG against phase II of *C. burnetii* was 18%. Seroprevalence between general practitioner practices ranged from 30% (48/160) at practice no. 4 in the Province of North-Brabant to 3% (4/147) in practice no. 10 in the province of Limburg (Table 1; Figure 1). A total of 4 general practitioner practices in the province of North-Brabant (nos. 1, 3–5), 1 general practitioner practice in the province of Friesland (no. 8), and 1 general practitioner practice in Limburg (no. 9) showed a seroprevalence rate >20%.

### Suspected Chronic Q Fever

A total of 9 patients (0.6% of participating patients, 3.2% of patients with a past *C. burnetii* infection) showed an IgG I titer >1:512 and were suspected of having chronic Q fever. Six practices had >1 patient suspected of having chronic Q fever (46%), and 3 of these practices had 2 patients suspected of having chronic Q fever. One practice in the Province of Limburg (no. 9) (Table 2; Figure 1) had 2 patients suspected of having chronic Q fever, which was 7.1% of seropositive patients in that practice. Another practice in Limburg (no. 12) had only 2 patients who had a past infection with *C. burnetii*, of whom 1 patient was suspected of having chronic Q fever.

### Patient Characteristics

We compiled patient characteristics for 3 groups: all participants, seropositive patients, and patients suspected of having chronic Q fever (Table 3). Patients suspected of having chronic Q fever were on average older and more often male than female. The most common risk factor among the participants was heart valve disorder (28%); this group also had a relatively high percentage of seropositive patients (22%), but no patients who had this risk factor were found to be suspected of having chronic Q fever. Within the risk factor groups, vascular disorders (ICPC code K99.01) (Table 1) had the highest percentage of seropositive patients (25%) and also the highest number (n = 4) suspected of having chronic Q fever. Among patients with an immunocompromised state caused by medication (ICPC code L04A) (Table 1), 13% were seropositive, and 3 patients were suspected of having chronic Q fever.

**Table 3:** Characteristics of all study participants, patients with a previous *Coxiella burnetii* infection, and patients suspected of having chronic Q fever, the Netherlands*

Characteristic	All participants, n = 1,642	Previous infection, IgG II titer >1:64, n = 289	Suspected of having chronic Q fever, IgG I titer >1:512, n = 9
Mean age, y	63	64	63
Age >60 y, % of total	66	65	78
Male sex, % of total	49	59	67
Risk factor, no.†			
Heart valve	460	103	0
Vascular	202	50	4
Other cardiovascular	105	20	1
Immunocompromised by illness	419	57	1
Immunocompromised by medication	445	59	3
Missing	135	18	0

*Corresponding International Classification of Primary Care codes for risk factor: heart valve, K83; vascular, K99.01; other cardiovascular, K73 and K71; immunocompromised by illness; B73, B74, B90, and D94; immunocompromised by medication, L04A. **†Some participants had multiple risk factors.**

### Diagnosis

Of the 9 participants suspected of having chronic Q fever, 8 went to a Q fever expert center for further medical examination. Chronic Q fever was ruled out in 4 patients, and 4 received a diagnosis of chronic Q fever (3 with probable chronic Q fever and 1 with proven chronic Q fever). One of the patients who had probable chronic Q fever did not receive treatment. The proven chronic Q fever patient had a vascular prosthesis, and the 3 probable chronic Q fever patients had a valve disorder, a vascular prosthesis, and an immunocompromised state caused by medication.

### Cost-effectiveness Analysis

**Figure 2:**
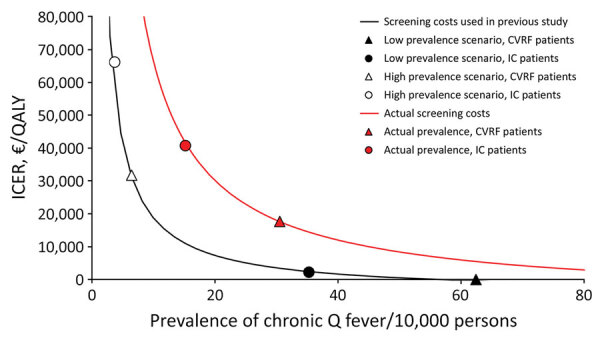
Relationship between the prevalence of chronic Q fever and incremental cost-effectiveness ratio of a screening program to detect chronic Q fever, the Netherlands, and screening costs for the program compared with a previously published analysis (*7*). Symbols on the line are based on a high-prevalence and low prevalence rate scenario as used in the previously published analysis and are based on actual prevalence rates found in this study. CVRF, cardiovascular risk factor; IC, immunocompromised; ICER, incremental cost-effectiveness ratio; QALY, quality-adjusted life year.

We determined the relationship between prevalence of chronic Q fever and the incremental cost-effectiveness ratio (ICER) and how the use of new data on screening costs and prevalence of chronic Q fever affected the model results (Figure 2). Because of the implementation costs of a stand-alone program in general practitioner practices and higher laboratory costs (the IFA is considerably more expensive than the ELISA), the actual screening costs per patient was 8 times higher (from €7 to €56; Table 4) than in the initial cost-effectiveness analysis. The 8-fold higher screening costs per person shifts the ICER considerably upwards, implying that the screening program becomes less cost-effective. The prevalence of chronic Q fever in our study, which is based on the 4 diagnosed chronic Q fever patients, was approximately in the middle of a low prevalence scenario and a high prevalence scenario considered in the previous cost-effectiveness analysis.

**Table 4:** Screening cost per patient in targeted screening program to detect chronic Q fever, the Netherlands, compared with previous cost-effectiveness analysis*

Item	Previous analysis	Actual
Diagnostic test	€7.26 (ELISA/IFA)	€25.00 (IFA)
Fee for trusted third party and general practitioner	NA	€24.36†
Logistics/coordination	NA	€4.30†
Start-up costs	NA	€2.69‡
Total	€7.26	€56.35

*Previous study in (*7*). IFA, immunofluorescence assay; NA, not applicable. †With a participation rate of 50%. ‡Total start-up costs of €135,000 divided by a previously estimated 71,000 eligible high-risk patients living in areas that had a high incidence of Q fever during the outbreak.

If one takes into account the actual screening costs and the prevalence rates, the ICER was estimated to be €17,643/quality-adjusted life year (QALY) gained for patients with a cardiovascular risk factor and €40,726/QALY gained for immunocompromised patients. Given that all detected chronic Q fever patients who had a cardiovascular risk factor were invited into the study because of a vascular or other cardiovascular disorder, the prevalence for this specific risk group was higher compared with that for all cardiovascular risk groups combined. When the analysis was stratified to vascular patients or other cardiovascular patients, screening became more cost-effective (ICER of €4,416/QALY gained).

## Discussion

This study shows that, even a decade after a large Q fever outbreak, targeted screening in high-risk groups living in previously highly affected regions still detects undiagnosed chronic Q fever patients. The cost-effectiveness analysis before the screening program considered a low-prevalence and high-prevalence scenario, given the high uncertainty around this parameter at that time (*8*). The prevalence of chronic Q fever in our study was within this range. However, the screening costs per patient were considerably higher than earlier anticipated. Nevertheless, screening remained cost-effective in certain high-risk groups, when an often-used cost-effectiveness threshold for preventive measures in the Netherlands of €20,000/QALY gained is applied.

The implementation of this targeted screening program was more labor intensive than expected, thereby increasing the costs. Although all general practitioner practices have a complete and up-to-date electronic file and use the same ICPC classification system, the computer-generated list of high-risk patients still needs manual checking. Eventually, we decided to provide active support for the general practitioners from a trusted third party, which increased costs.

In half of the general practitioner practices, we found a seroprevalence of antibodies against phase II of *C. burnetii* >20%, and 1 practice reported a seroprevalence of 30%. Although participants are not randomly selected (most invited persons were elderly and the willingness to participate in the screening program might have been influenced by the considered risk for exposure), such a high prevalence of person with evidence of a past infection is remarkable. Previous population-based studies from the southern region of the Netherlands found seroprevalences of 2%–14% (*12*). Particularly of interest was the high seroprevalence of 28% in a general practitioner practice located in the northern region of the Netherlands, where hardly any acute Q fever cases were reported during the outbreak in 2007–2010. These figures show the complex spatial‒temporal dynamics in exposure, infection, and disease.

Our study’s first limitation is that, of the 9 participants suspected of having chronic Q fever, only 4 received a definite diagnosis. Antibody titers might vary for patients (*13*), and the IFA is known to have a high measurement uncertainty. Also, half of the invited persons did not participate in the screening program, and it is unknown whether this factor led to missed chronic Q fever patients. The COVID-19 pandemic, which was ongoing at the time, might have played a role because attending health services for nonacute problems were discouraged.

Of the 4 chronic Q fever patients, 3 (75%) received a probable and 1 (25%) a proven diagnosis. In the previous cost-effectiveness analysis, we based the ratio of probable and proven chronic Q fever patients on the national chronic Q fever database (31% probable and 69% proven) (*14*). Because patients who have probable chronic Q fever have a lower risk for complications and death than patients who have proven chronic Q fever (*2*), using the ratio from our study would make the screening program less cost-effective than the ratio from the database. Other hospital-based screening studies among high-risk patients also found a relatively higher proportion of probable chronic Q fever patients than in the database (*5*,*7*). A potential explanation might be that some probable patients eventually progress into proven patients before they are given a diagnosis during regular care. Therefore, we maintained the use of the ratio from the database in the updated cost-effectiveness analysis because the number of patients detected in this first phase of the screening program is still limited and might be coincidental.

In a continuation of this screenings program, some suggestions for modification to improve its (cost-)effectiveness can be made. First, the ICPC code K83 (nonrheumatic valve disease) could potentially be omitted because this disease was the largest risk group in the program, but without any suspected chronic Q fever patient. This particular ICPC code comprises a heterogeneous group of heart valve disorders, and perhaps this code is not representative for those heart valve patients that are at increased risk for chronic Q fever, as found in a previous screening study (*7*). Another explanation could be that heart valve patients at increased risk for chronic Q fever might already be represented in the category of other cardiovascular disorders (K73 and K71) (Table 1) or have a combined vascular and heart valve disorder. Second, the high-risk regions could be tailored to smaller areas near goat farms that were affected by Q fever‒induced abortion waves and where airborne transmission is likely to have occurred. In our study, seroprevalence and the number of suspected chronic Q fever patients differed considerably between regions, and general practitioner practices that had the highest seroprevalence and with >1 suspected chronic Q fever patient were near an infected farm (<5 km). A previous study from the Netherlands also indicated that the seroprevalence of antibodies against *C. burnetii* is strongly correlated with proximity to a goat farm, but not with Q fever incidence during the outbreak (*12*).

Results of the present screening program were communicated to the Ministry of Health, which subsequently submitted a report on the state of affairs regarding Q fever to the Parliament of the Netherlands on September 29, 2021 (*15*). If the screening program would be continued, it should be better embedded in the regular general practitioner care by creating more awareness among general practitioners and their patients of the risks of chronic Q fever and the possibilities of selecting and screening high-risk patients through the general practitioner electronic records. The active support of a third party should not be necessary and general practitioners should be able to integrate this into their daily practice with a clear and concise instruction, which needs to include the lessons learned during the early phase of this program.
